# Complete Genome Sequence of Bacillus subtilis BYS2, a Strain with a Broad Inhibitory Spectrum against Pathogenic Bacteria

**DOI:** 10.1128/MRA.00803-21

**Published:** 2021-09-16

**Authors:** Mengjiao Guo, Donghui Liu, Tongjie Chai

**Affiliations:** a Jiangsu Co-innovation Center for Prevention and Control of Important Animal Infectious Diseases and Zoonoses, College of Veterinary Medicine, Yangzhou University, Yangzhou, Jiangsu, China; b College of Animal Science and Veterinary Medicine, Shandong Agricultural University, Tai’an City, Shandong Province, China; University of Rochester School of Medicine and Dentistry

## Abstract

Bacillus subtilis BYS2 is a strain with a broad inhibitory spectrum against pathogenic bacteria. In the current study, we report the complete genome sequence of Bacillus subtilis BYS2. The chromosome of BYS2 (4,030,791 bp; G+C content, 43.88%) contained 3,914 protein-encoding genes, with 86 tRNAs, 30 rRNAs, and 5 noncoding RNAs (ncRNAs).

## ANNOUNCEMENT

Bacillus subtilis has been successfully used as a probiotic, which has benefits for humans and animals for growth promotion and disease prevention (1, 2). B. subtilis BYS2 was isolated from a soil sample collected at a depth of 10 cm beneath the surface of Mount Tai in Tai’an (Shandong Province, China). The soil sample was suspended in normal saline and cultured at 80°C for 1 h. Then, the culture was spread onto nutrient agar and incubated at 37°C for 24 h. B. subtilis BYS2 was identified by the colony morphology (showing a typical volcano shape), Gram stain result (positive), biochemical tests, and 16S rRNA sequencing. *In vitro*, BYS2 exhibited excellent antimicrobial activity against pathogenic bacteria (3). We have demonstrated that diets containing BYS2 can improve immunity and disease resistance in rabbits (3). BYS2 can also improve growth performance, immune responses, and disease resistance against Escherichia coli in chickens (4). Here, we report the complete genome sequence and annotation of B. subtilis BYS2.

B. subtilis BYS2 was grown in nutrient broth medium at 37°C for 12 h. Genomic DNA was extracted using the bacterial genomic DNA kit (CWBio, Beijing, China). The genomic DNA sample was quantified using a NanoDrop 2000c spectrophotometer (Thermo Scientific, MA, USA). DNA was sheared using a g-TUBE device (Covaris, MA, USA) to target 20-kb fragments. Then, more than 6 μg sheared and concentrated DNA (optical density ratio at 260/280 nm [OD_260/280_] range, ∼1.8 to 2.0) was utilized for size selection using the Blue Pippin system (Sage Science, Beverly, MA, USA). A 20-kb library was constructed using the SMRTbell template prep kit v2.0 (PacBio, CA, USA) and sequenced on a Sequel II system (v2.0 chemistry; Sequel II v8.0 system software; 15-h movie) (5). A total of 150,292 polymerase reads were obtained. Reads with a length less than 100 bp or average quality less than 0.80 were removed. After quality filtering, 83,099 reads were assembled *de novo* using the Hierarchical Genome Assembly Process (HGAP) v2.3.0 with default parameters (6). The genome coverage was 249.02×, and the *N*_50_ value was 17,711 bp. The preliminarily assembled sequence was automatically assembled into a ring based on the overlap using Circlator v1.5.5 with the minimus2 circularization pipeline. Then, the genome was rotated using the fixstart method in Circlator (7). Automated gene annotation of the BYS2 genome sequence was carried out using the NCBI Prokaryotic Genome Annotation Pipeline (PGAP) v5.2 with default parameters during upload to GenBank (8). The total repeats of the circular genome were predicted using RepeatMasker v4.1.2 (http://www.repeatmasker.org/). IslandViewer v4 (http://www.pathogenomics.sfu.ca/islandviewer/) was used to predict the gene islands (9). The secondary metabolic gene clusters of BYS2 were predicted using antiSMASH v6.0.1 (10). In addition, default parameters were used for all software unless otherwise specified. The genome consists of a single chromosome of 4,030,791 bp (G+C content, 43.88%). Our analysis predicted 3,914 protein-encoding genes. There are 4 types of repeat sequences, including short interspersed nuclear elements (SINEs), long interspersed nuclear elements (LINEs), long terminal repeat (LTR) elements, and DNA elements. A total of 22 gene islands were predicted. Furthermore, 86 tRNA genes, 30 rRNA genes (5S rRNAs, 10; 23S rRNAs, 10; 16S rRNAs, 10), and 5 noncoding RNAs (ncRNAs) were identified. No plasmid-related sequences were detected (Table 1). The *albA*, *albB*, *albC*, *albD*, and *albE* genes, which are involved in bacteriocin production and immunity, were found (11). Moreover, several types of secondary metabolic gene clusters were identified, including 3 nonribosomal peptide synthases (NRPS), 1 lantipeptide, 2 terpenes, 1 type III polyketide synthase (T3PKS), 1 sactipeptide, 1 *trans*-acyltransferase polyketide synthase (*trans*-AT-PKS), and 1 other containing a secondary metabolite-related protein that does not fit into any other category.

**TABLE 1:** Genome features of Bacillus subtilis BYS2

Genome feature	Value
GC content (%)	43.88
Total length (bp)	4,030,791
No. of CDSs	3,914
No. of tRNAs	86
No. of rRNAs	30
No. of ncRNAs	5
No. of gene islands	22
No. of secondary metabolic gene clusters	7

### Data availability.

The genome sequence of B. subtilis BYS2 was deposited in GenBank under the accession number CP074571.1. The BioProject accession number is PRJNA726833, and the BioSample accession number is SAMN19091144.
